# Usability of a barcode scanning system as a means of data entry on a PDA for self-report health outcome questionnaires: a pilot study in individuals over 60 years of age

**DOI:** 10.1186/1472-6947-6-42

**Published:** 2006-12-21

**Authors:** Patrick Boissy, Karen Jacobs, Serge H Roy

**Affiliations:** 1 Research Centre on Aging, Sherbrooke Geriatric University Institute, Sherbrooke, Canada; 2 Université de Sherbrooke, Department of Kinesiology, Sherbrooke, Canada; 3 Boston University, Sargent College of Health and Rehabilitation Sciences, Department of Occupational Therapy and Rehabilitation Counseling, Boston, USA; 4 Boston University, NeuroMuscular Research Center, Boston, USA

## Abstract

**Background:**

Throughout the medical and paramedical professions, self-report health status questionnaires are used to gather patient-reported outcome measures. The objective of this pilot study was to evaluate in individuals over 60 years of age the usability of a PDA-based barcode scanning system with a text-to-speech synthesizer to collect data electronically from self-report health outcome questionnaires.

**Methods:**

Usability of the system was tested on a sample of 24 community-living older adults (7 men, 17 women) ranging in age from 63 to 93 years. After receiving a brief demonstration on the use of the barcode scanner, participants were randomly assigned to complete two sets of 16 questions using the bar code wand scanner for one set and a pen for the other. Usability was assessed using directed interviews with a usability questionnaire and performance-based metrics (task times, errors, sources of errors).

**Results:**

Overall, participants found barcode scanning easy to learn, easy to use, and pleasant. Participants were marginally faster in completing the 16 survey questions when using pen entry (20/24 participants). The mean response time with the barcode scanner was 31 seconds longer than traditional pen entry for a subset of 16 questions (p = 0.001). The responsiveness of the scanning system, expressed as first scan success rate, was less than perfect, with approximately one-third of first scans requiring a rescan to successfully capture the data entry. The responsiveness of the system can be explained by a combination of factors such as the location of the scanning errors, the type of barcode used as an answer field in the paper version, and the optical characteristics of the barcode scanner.

**Conclusion:**

The results presented in this study offer insights regarding the feasibility, usability and effectiveness of using a barcode scanner with older adults as an electronic data entry method on a PDA. While participants in this study found their experience with the barcode scanning system enjoyable and learned to become proficient in its use, the responsiveness of the system constitutes a barrier to wide-scale use of such a system. Optimizing the graphical presentation of the information on paper should significantly increase the system's responsiveness.

## Background

Throughout the medical and paramedical professions, self-report health status questionnaires are used to gather patient-reported outcome (PRO) measures. PRO data from self-report health status questionnaires are collected at the point of care in clinical and research settings to help guide patient assessment, diagnosis and care planning, and to track patients' progress [1]. Dedicated personnel generally administer survey instruments during interviews or have patients complete questionnaires. Although different approaches are used to gather the information (i.e. self-report, use of a proxy, interview), paper-and-pencil is still the most common method of data entry used by clinicians and researchers using self-report based health status questionnaires. Traditionally, information is converted into a format suitable for computerized quantitative data analysis, either by manual data entry (single or double key punching) or scanner technology. This can severely burden clinicians and researchers with unmanageable quantities of paperwork, compromise the accuracy of the information obtained, delay information processing and tranfer, and increase associated research and care management costs. Errors can also occur when completing the questionnaires at the point of data entry, transcribing the data for digitization to a computer database, or processing the information for tabulating scores and generating reports [2]

Electronic capture of PRO data (ePRO) from self-report health status questionnaires using computers is seen as a solution to these problems. While ePRO is mostly used in the context of clinical trials under stringent controlled conditions [3], the emergence of mobile computing platforms such as PDAs, tablets or laptop PCs has expanded its use in numerous clinical and health service research applications [4-10]. Among these platforms, pen entry on PDAs is the most widely used and tested method in medical fields. However, the usability of PDA devices with older adults in the context of data entry for self-report health outcome questionnaires has not been studied extensively. Usability is defined as the extent to which a product can be used by specified users to achieve specific goals effectively, efficiently, and with satisfaction in a specified context of use [11]. Usability is a multidimensional attribute used in the study of human-machine interaction to assess the ease with which a user can learn to operate, prepare inputs for, and interpret outputs of a system or component. PDAs have relatively small displays with limited resolution and data entry is accomplished primarily through the use of a stylus and touch screen. Because visual acuity, contrast-sensitivity function, and fine motor skills decrease with age, the usability of the user interface found in PDAs and the modes of data entry on these devices are not appropriate for most older adults [12]. They work relatively well in the hands of experienced able-bodied young people but often fail when one of these devices is given to an older adult with limited computer skills, poor eyesight, and imperfect hand-eye coordination or movement disorders. The objectives of this study were thus to develop an alternative electronic data entry method for self-report questionnaires and explore its usability with older adults in the context of collecting outcome measures.

## Methods

### Barcode entry system

**Figure 1:**
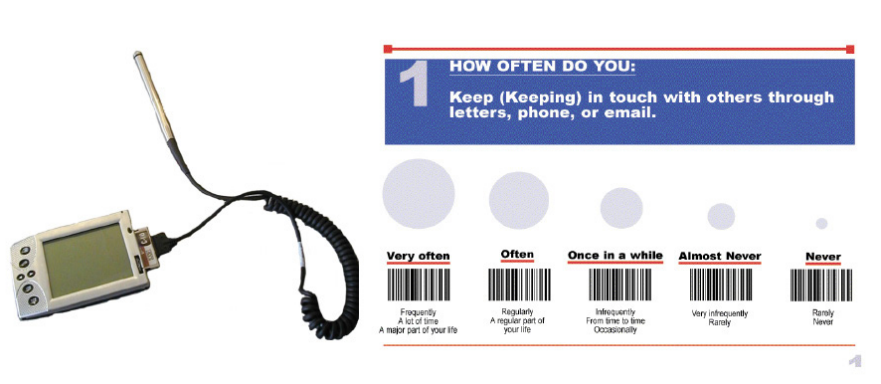
**Overview of proposed system to collect outcome data electronically**. A mobile computer (top left panel) accepting flash card adapters and running the Palm OS is connected to a barcode wand scanner to input outcome data. The survey questions and their answer fields are presented on paper (top right panel). Barcodes are associated with specific answer fields. The layout can be adjusted (type face, presentation of information etc.). Once collected electronically on the mobile computer, the information can be transferred to a PC database.

The components used for the data collection system are presented in Figure 1. The hardware components of the system comprise a mobile computer (Handera 330, Handera) running the Palm™ operating system, a flash card barcode wand scanner (Bar Wand CF Card, Socket) and a text-to-speech synthesizer (DoubleTalk PC text-to-speech synthesizer, RC system) with an audio output speaker module. The software components comprise a modular data acquisition platform running on the mobile computer to record data inputs from the users, a conduit to an ODBC driver and a PC database.

In the proposed system, questions and answers from health status questionnaires are programmed and then compiled in an electronic form with data entry input masks specific to the data entry structure of a given questionnaire. A data entry input mask is a representation of answer fields that a user will activate through a specific action (i.e. checking a box corresponding to a given answer). Data entry input masks are linked to unique identifiers in a database stored on the mobile computer. Electronic forms can be a single questionnaire or multiple questionnaires as part of a library of health outcome measures. Information from health outcome questionnaires is presented on paper where each question and potential answer is assigned an 8-digit barcode. Upon successful scanning of a barcode for a given answer to a specific health outcome question, the answer field is activated on the data capture mask on the mobile computer data acquisition platform and the corresponding answer is read out loud on a speaker by the text-to-speech voice synthesizer. The system can be used by clinical personnel during interviews or by the patients themselves with relatively little training or supervision. After completion of the health outcome questionnaire, a report (scores or norm) can be generated and printed or transmitted by email to a third party, and the raw data transferred to a PC relational database.

### Usability testing scenario

The usability of the barcode scanning system as a data entry method for self-report questionnaires was tested on 24 older adults recruited from the community. All subjects provided written informed consent prior to participating in the study. The study was approved by the Charles River Institutional Review Board (IRB) of Boston University. A usability testing scenario was established. Instructions (verbal and written) and interactions between participants and research staff were standardized to make sure that participants received the same amount of attention and were exposed to the same conditions. Prior to the usability test, a member of the research staff provided instructions and demonstrated the use of the barcode scanner to the participants. This tutorial was followed by a practice session, which required the participants to independently scan a series of barcodes (n = 25) positioned horizontally on paper. Participants completed the practice session in less than 3 minutes.

Upon completion of the tutorial and practice session, participants were assigned to a usability test sequence where they had to complete two sets of 16 questions from the Late Life Function and Disability Instrument (LLFDI) using the bar code wand scanner for one set and a pen for the other. The LLFDI is an outcome survey on activity and participation that assesses physical functioning and disability in terms of frequency, limitation and difficulty in performance of life tasks [13,14]. Survey questions from the LLFDI in the barcode entry tasks were presented individually on paper with answer fields represented as bar codes which participants scanned using the bar code wand (see Figure 1). For pen entry, the LLFDI survey questions were presented using the traditional paper representation of the survey. The order of data entry method (barcode wand scanner vs. pen) was randomized across participants. Upon completion of the two sets of questions, participants were given the choice of selecting one of the data entry methods to complete a third set of questions from the LLFDI survey. Because receptiveness to the use of a computerized electronic assessment can be influenced by computer anxiety and prior experience [15,16], the participants' attitudes toward computers were evaluated using the Attitudes Toward Computer Questionnaire (ATCQ) originally developed by Bear and colleagues [17] and validated on older adults [18,19]. The questionnaire consists of 48 statements that evaluate attitudes and beliefs about computers on 7 dimensions (comfort, efficacy, gender equality, control, dehumanization, interest and utility). Participants express their level of agreement with each statement using a 5-point Likert scale. Scores are computed on each subscale.

### Usability measures

Usability can mean different things to different people [20]. In this study we chose a performance-based approach [21] to evaluate the usability of barcode scanning in terms of task times and areas of difficulty during a test scenario. This was supplemented by an interview with a questionnaire. Participants were videotaped through a one-way mirror with a camera set up to capture video sequences of the subjects while they completed the survey. Participants were informed that they were being videotaped. The camera system generated a continuous time code on tape so that events could be time-stamped for later analysis using an observation grid. No assistance was provided to the participants as they completed the survey. Usability was assessed through video analysis of the participants during completion of each task in the usability test scenario and through directed interviews with a usability questionnaire. Upon completion of each task in the usability test scenario, the interviewer came back into the room to interview the participants on the learnability of using a barcode scanner for data entry and their subjective satisfaction with the barcode scanning system, and to compare the experience of using the bar code scanner with pen entry when filling out a outcome questionnaire. Statements pertaining to these themes were formulated and adapted from existing usability testing literature [22]. The questions are given in Appendix 1 (see additional file 1). Participants indicated their level of agreement on a 5-point Likert scale. Responses to each question were assigned numerical values from 0 (for responses corresponding to "completely disagree") to 100 (for responses corresponding to "completely agree"). Mean scores for each section of the questionnaire were computed per participant. Retrospective analysis of the tapes used the time codes to compute the total time to complete each section of the survey using barcode entry or pen entry. For each survey question answered using the barcode system, the number of scans needed by the participants to successfully input their response in the system was tabulated. First scan success rate expressed as a percentage was computed individually as the number of questions that a participant successfully answered with one pass of the barcode scanning system out of a total of 16 questions. The barcode locations on paper associated with unsuccessful first scans were also tabulated. Frequency counts of scanning error barcode locations were computed from group data.

## Results

### Participants' characteristics

Older adults (n = 24) were recruited from the community using a list of participants previously enrolled in studies at Boston University's Rehabilitation Research and Training Center on Measuring Rehabilitation Outcomes. The usability testing scenario and interviews were completed within one hour for all participants. No technical problems with the equipment occurred and the majority of participants (75%) chose the barcode scanner to complete the third series of survey questions. The participants' sociodemographic characteristics and scores on each of the scales of the adapted version of the ATCQ are presented in Table 1 and Table 2, respectively.

**Table 1:** Sociodemographic characteristics of the participants

	**Categories**	**Frequency**	**% of sample**	**% of USA**
**Age**	60–69	8	33	44
	70–79	9	37	35
	80–89	6	25	17
	90 et +	1	4	4

**Gender**	Male	7	29	40
	Female	17	71	60

**Race**	White	16	67	84
	Black	6	25	8
	Hispanic	2	8	5

**Education**	Some High School	2	8	43
	High School Diploma	5	21	29
	Some College	9	37	15
	Bachelor Degree	4	17	7
	Graduate Degree	4	17	4

**Table 2:** Results on attitudes toward technology surveys

**Variables**	**Means (%)**	**Std (%)**	**Range (%)**
**Comfort**	66	13	44–92
**Efficacy**	72	11	55–92
**Gender bias**	71	10	48–88
**Control**	66	13	44–88
**Dehumanization**	53	11	40–76
**Interest**	81	9	65–99
**Utility**	74	11	56–96

The participants (7 men, 17 women) ranged in age from 63 to 93 years with an mean of 76 years of age. The majority of participants were college-educated (65%) and some (17%) had a graduate degree. The ethnic background of the participants was predominantly Caucasian (16 out of 24) with 6 African American and 2 Hispanic participants. Overall, participants showed a favorable attitude toward technology prior to using the system. On the ATCQ, higher numbers represent more favorable attitudes toward computers. For example, "keen on the subject of technology" scored 81% ± 9% (mean +/- SD) on the interest scale, "positive outlook on its utility" scored 74% ± 11% (mean +/- SD) on the utility scale, and "comfortable with its use" scored 66% ± 13% (mean +/- SD) on the comfort scale.

### Time to complete survey questions

**Figure 2:**
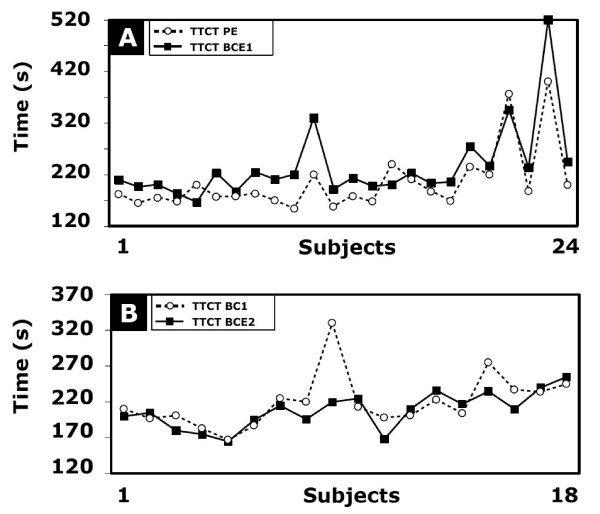
**Individual results comparing the total time to complete task (TTCT)**. **A) **Pen entry (PE) on paper vs. first round of bar code entry (BCE) after a brief tutorial and practice session (n = 24). **B) **First round of bar code entry after brief tutorial and practice session vs. second round of data entry using barcode entry for participants who chose to continue with the barcode system (n = 18). Mean time and standard deviation for TTCT with BCE was 235 ± 74 seconds vs. 204 ± 61 seconds for TTCT with PE in the first round of data entry. For subjects who completed 2 rounds of data entry using the barcode system, mean time and standard deviation for TTCT with BCE on the first round was 219 ± 37 seconds vs. 191 ± 25 seconds for TTCT with BCE in the second round of data entry.

The time to complete survey questions using either barcode entry or pen entry is illustrated in Figure 2. Participants completed the 16 survey questions faster when using pen entry (20/24 participants). Mean response time with the barcode scanner was 31 seconds longer than traditional pen entry for a subset of 16 questions (t(23) = 4.16, p = 0.001). Mean response time decreased from 31 seconds to 16 seconds for participants who answered the third series of questions using barcode entry (t(17) = -2.63, p = 0.018). No significant differences (t(17) = -1.6, p = 0.13) were observed between each series of questions answered with barcode entry (BCE1 vs BCE2).

### First scan success rates and scan error locations

**Figure 3:**
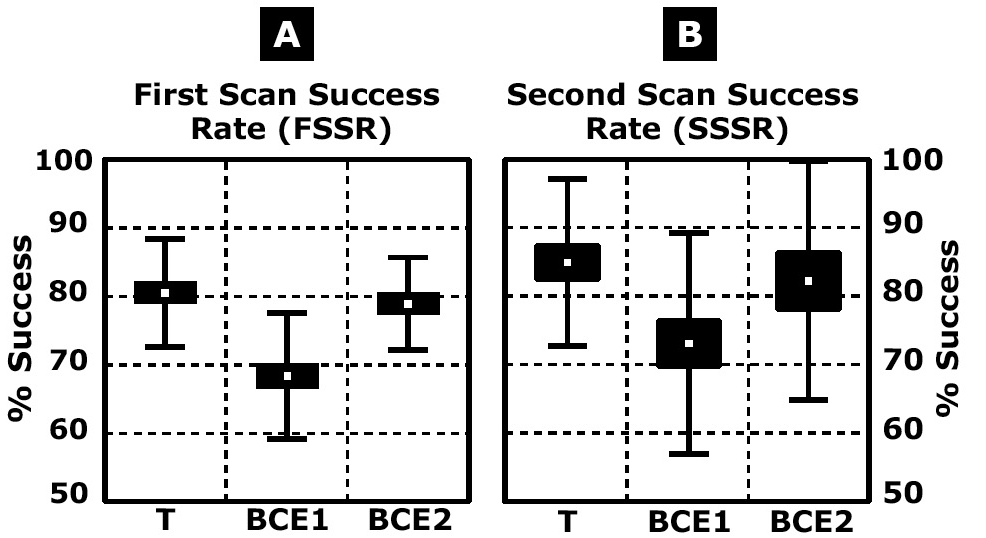
**Scan success rates when using the bar code entry system during the tutorial/practice (T), first round of bar code entry (BCE1) and second round of bar code entry (BCE2)**. **A) **Mean and standard deviation of first scan success rate expressed as a % of the questions answered across participants (n = 24). **B) **Mean and standard deviation of second scan success rate for unsuccessful first scan (expressed as a % of the questions answered) across participants (n = 24).

The responsiveness of the scanning system for the practice barcode entries, first round of barcode entries (BCE1), and second round of barcode entries (BCE2), is compared in Figure 3, where first and second scan success rates are provided for the 16 questions. The BCE1 success rate was generally high, with an mean success rate of 68%. Mean first scan success rates were higher during the practice session than when completing the survey questions (t(23) = 7.82, p = 0.001). For those who did not succeed on the first try, a second scan was enough to achieve success in 75% of the cases, for an overall mean success rate of 90% when allowing for up to 2 scans. No participant required more than 3 scans on any of the survey questions. Mean first scan success rates increased significantly from 68% in the first round of barcode entry to 79% (t(17) = 6.19, p = 0001) for those completing a second round of barcode entry (BCE2).

**Figure 4:**
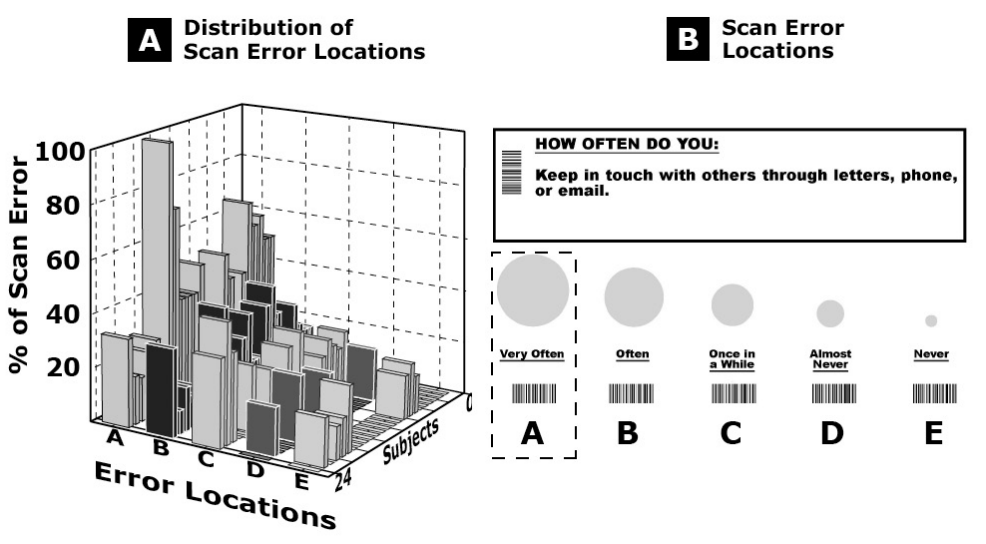
**Scan errors**. **A) **Frequency distribution; and **B) **Location of first scan errors on paper. Most first scan errors occurred when scanning barcodes associated with answers positioned on the far left of the paper.

The distribution of scanning errors during the first round of barcode entry (i.e. barcodes that were unsuccessfully scanned on the first scan) relative to the location of the responses on the paper version of the survey is presented in Figure 4. It should be noted that only 2 participants were left-handed. The scanning errors were associated mostly with barcodes located on the far left of the paper with, 40% ± 21% (mean +/- SD) of the recorded errors for location A, 20% ± 11% (mean +/- SD) for location B, 12% ± 14% (mean +/- SD) for location C, 3% ± 7% (mean +/- SD) for location D, and 5% ± 8% (mean +/- SD) for location E. Scanning errors in location A were significantly greater than other locations (F(115) = 359, p = 0.001, pairwise contrast analysis p = 0. 001).

### Usability questionnaire

**Figure 5:**
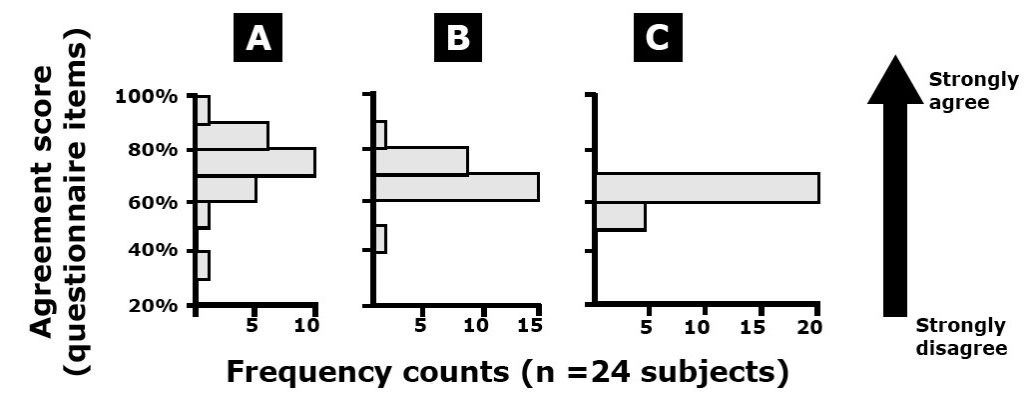
**Subjective evaluation of barcode scanning experience as measured by usability questionnaire (see Appendix 1) administered after tutorial/practice, first round of data entry, and second round of data entry**. Frequency distribution (n _total _= 24) of mean scores on: **A) **Learnability of using a barcode scanner for data entry; **B) **Satisfaction after using the barcode scanner; and **C) **Experience with bar code scanner vs. pen entry. A mean score higher than 60% indicates agreement with statements.

Summary results of the usability questionnaire, expressed as frequency distributions for mean scores computed from questions specific to learnability and the subjective satisfaction experience of using the bar code scanner, are illustrated in Figure 5. Participants' responses on statements pertaining to learnability suggest that learning and mastering the use of the barcode scanning system was easy and that participants felt they could achieve proficiency with the system within the context of the tutorial and practice sessions offered prior to the usability testing scenario. Only 2 participants out of the 24 had a negative perception of the learnability of the barcode system (i.e. mean score lower than 60% indicating disagreement with statements; mean +/- SD score for the group was of 71% ± 12%). Results on the subjective satisfaction scale indicate that participants were comfortable with the system, did not find it too complex or cumbersome, and generally felt confident when using it. Mean scores on the subjective satisfaction scale after completing the first round of data entry using the barcode scanning system range from 44% to 85% with an mean +/- SD of 69% ± 7%. Only one subject had a negative perception on the subjective satisfaction scale. This was the same individual who had an unfavorable learnability score. Mean scores on statements comparing barcode entry to pen entry suggest that participants would not favor one data entry method over the other with respect to enjoyment, ease of use and effectiveness of completing an outcome questionnaire. Mean scores were distributed around the 60% mark and varied from 50% to 68% with an mean +/- SD of 62% ± 6%. Out of the 24 participants, 7 had a negative perception on the comparative scale.

## Discussion

ePRO of self-report health outcome questionnaires has been suggested as a data entry method to improve the data collection process by reducing paperwork and administrative costs; to increase data accuracy by enforcing collection of more complete information and eliminating redundant data entry; and to improve the information flow by reducing data backlog and providing access to previously unavailable information [3]. While the psychometric qualities of self-report health status measures acquired through pen entry on a personal digital assistant (PDA) have been shown to be similar to those using pen-and-paper instruments [8,23,24], evidence regarding the usability of this type of approach with older adults is lacking as the majority of usability studies on mobile computing devices in healthcare have focused on the use of PDAs by nurses and physicians, not by patients or research subjects. Usability issues when using PDAs as tools to collect information typically include screen size, handwriting recognition problems and data entry mechanisms [25]. An ePRO system combining barcode scanning with a voice synthesizer was developed and tested to address such difficulties. The concept behind the system is to use barcode scanning of answer fields from survey questions to input data electronically on a database residing on a PDA. The usability of a data entry system based on barcode scanning for electronic data capture of self-report health outcome questionnaires was evaluated in older adults living in the community.

Participants were able to use the system to answer questions from an outcome questionnaire with limited instructions and no supervision. While the time to complete the survey questions using the barcode entry system was statistically longer than the time needed to complete the questions using pen entry, the difference can be considered marginal for real life applications, and is certainly offset by the time saved in avoiding the need for subsequent manual input of the data into a computer, which would likely be required when processing the pen entry data. Barcode scanner responsiveness was less than perfect, with approximately one-third of first scans requiring a rescan to successfully capture the data entry.

The lack of responsiveness of the system can be explained by a combination of factors such as the location of the scanning errors, the type of barcode used as an answer field on the paper version, and the optical characteristics of the barcode scanner. A high percentage of first scan errors was located on answer fields on the far left of the paper, or more specifically for answer fields closest to the edge of the paper. Video analysis of first scan errors showed that unsuccessful scans were associated with starting the scanning motion too close to the printout of the barcode on the paper or putting too much pressure on the paper, thus slowing the scanning motion considerably. For barcodes to be scanned effectively, users must use a fluid motion from start to finish and start their scan at least one cm before the beginning of the barcode. The optical sensitivity of the barcode scanner in distinguishing blank space from space occupied by the barcode may therefore be a factor when considering ways to reduce first scan errors in future applications. Moreover, the precision of the positioning of the barcode scanner and the fluidity of the scanning motion were also affected by the hand dominance of the participants (92% righthanded). Answer fields positioned on the far left required more complex coordination of shoulder, elbow and wrist joints to position the barcode wand scanner and perform the scanning motion. However, it would seem that the need to use a second pass to successfully scan the chosen answer field did not have much impact on the time taken to complete the tasks and the participants' subjective satisfaction with the system.

The results presented in this study offer insights regarding the feasibility, usability and effectiveness of using a barcode scanner with older adults as an electronic data entry method when completing patient-reported outcome questionnaires on a PDA. This was an exploratory study in a relatively small sample (n = 24) where the usability of a novel approach to data entry was assessed under conditions that approximated a real life clinical context. The sample size proved sufficient to identify barriers and obstacles that should be considered when designing and optimizing such a system for wide-scale use under the proposed specific application. Furthermore, while participants in this study found their experience with the barcode scanning system enjoyable and learned to become proficient in its use quite quickly, their educational background and generally positive attitudes toward technology could have introduced a positive bias in their evaluation of this device. In fact, the participants were highly educated and well above the average for adults, according to U.S. population statistics.

When this study began, there were no commercially available systems designed specifically for the application we studied. A barcode scanning system with text-to-speech feedback combined with PDA technology was configured based on the consideration that such technologies could complement one another in providing greater flexibility and usability compared to paper-based systems or pen entry on a PDA when completing health status questionnaires. The barcode technology gets around the limitations associated with PDA screens and the use of a stylus. The flexibility of using a traditional paper-based medium to present information offers many possibilities for users to adapt such forms relatively easily using word processing and graphical software packages, as well as the ability to duplicate them on demand using a variety of print media. The paper layouts on which the barcodes are presented can be adapted to the user's characteristics (young, old, literacy, and linguistic backgrounds) or the context of use (interview or patient-entered information) through changes in the language of the text, graphical representation, color, or font size. The electronic data capture infrastructure programmed on the PDA is hard-coded but it is written in a way that allows the user to customize the method of data entry into the system. The use of paper versions and a data entry interface such as the barcode wand scanner make interactions between the ePRO system and the user more natural. The barcode wand scanner is similar in shape to a pen and can be easily manipulated with less dexterity than when writing with a pen. The use of a barcode scanner linked to a text-to-speech synthesizer provides dual feedback on both scanning success and accuracy of the item scanned. The features can be activated or not at the user's discretion and could also be used for communication purposes in people with cognitive difficulties or the inability to communicate quickly through the written word.

## Conclusion

The results presented in this study offer insights regarding the feasibility, usability and effectiveness of using a barcode scanner with older adults as an electronic data entry method on a PDA. With limited instruction and practice, participants successfully used the barcode scanner to answer survey questions from a self-report outcome questionnaire without assistance. While participants in this study found their experience with the barcode scanning system enjoyable and learned to become proficient in its use, the responsiveness of the system constitutes a barrier to wide-scale use of such a system. Optimizing the graphical presentation of the information on paper should significantly increase the system's responsiveness. Further testing on a larger sample is needed to address performance and reliability issues and ultimately compare the effectiveness of this method with other means of collecting information electronically from self-report health outcome questionnaires. Recent developments in pen-driven computing such as Tablet PCs (cost considerations aside) have provided fertile ground for new applications of ePRO.

## Competing interests

The author(s) declare that they have no competing interests.

## Authors' contributions

PB participated in the design and data collection of the study, performed the data analysis and drafted the manuscript. KB participated in the design and data collection and helped to draft and revise the manuscript. SR participated in the design and coordination of the study and helped to draft and revise the manuscript. All authors read and approved the final manuscript.

## Pre-publication history

The pre-publication history for this paper can be accessed here:



## Supplementary Material

Additional File 1 Appendix 1. Usability questionnaire. 5-point Likert scale ssability questions that were asked after the participants used the barcode system. Click here for file
