# Supplementary material for: Usability of a barcode scanning system as a means of data entry on a PDA for self-report health outcome questionnaires: a pilot study in individuals over 60 years of age
Source: BMC Med Inform Decis Mak. 2006 Dec 21;6:42. doi: 10.1186/1472-6947-6-42 (PMC1769483; doi:10.1186/1472-6947-6-42)
Supplement: Additional File 1 — Appendix 1. Usability questionnaire. 5-point Likert scale ssability questions that were asked after the participants used the barcode system. [file 1472-6947-6-42-S1.pdf]

APPENDIX 1. Usability questionnaire

I am going to read to you several statements that pertain to the task you just completed. I want you to indicate by pointing on the scale shown on this sheet how much you agree or disagree with each statement, where 1 means you strongly disagree with the statement and 5 means you strongly agree with the statement

1. Interview questions on learnability of barcode system after 3-minute tutorial

1. Learning to use the scanner system was not easy for me (reverse scoring).
2. It was easy for me to become skillful at using the scanner system.
3. I found the scanner system easy to use.
4. I found the scanner system to be flexible to interact with.
5. I found that it was easy to get the system to do what I wanted it to do.
6. I would imagine that most people would learn to use the scanner system very quickly.

2. Interview questions on subjective satisfaction with barcode entry system after first round of barcode entry

1. The scanner system is pleasant to use.
2. Using the scanner system is effortless.
3. I found the scanner system cumbersome to use (reverse scoring).
4. I found the scanner system easy to use.
5. The scanner system does everything I would expect it to do.
6. The scanner system is unnecessarily complex (reverse scoring).
7. I can use the scanner system without assistance to fill out the questionnaire.
8. The scanner system is fun to use.
9. The scanner system works the way I want it to work.
10. I can't remember very easily how to use the scanner system (reverse scoring).
11. I did not feel very confident using the scanner system (reverse scoring).
12. It was easy to read the instructions and questions on the questionnaire.
13. It was easy to understand the instructions and questions on the questionnaire.
14. It was simple to fill out the questionnaire.
15. I felt comfortable filling out the questionnaire.
16. I am satisfied with using the scanner system to fill out the questionnaire.

3. Interview questions comparing barcode entry to pen entry after third round of data entry

1. Using the scanner to fill out questionnaires would make it easier for me than using a pen.
2. Using the scanner to fill out questionnaires would make it more enjoyable for me than using a pen.
3. Using the scanner system to fill out questionnaires would keep me more focused on the task than using a pen.
4. Using the scanner system to fill out questionnaires would enable me to complete this task more quickly than using a pen.
5. Using the scanner system would enhance my effectiveness in completing questionnaires.
